# IMACulate(DE3), an *E. coli* Strain for High Purity His‐Tagged Protein Purifications

**DOI:** 10.1002/bit.70244

**Published:** 2026-05-19

**Authors:** Jia Q. Truong, Dingyi Yu, Nan Hao, Chris Langendorf, Keith. E. Shearwin, Jessica K. Holien

**Affiliations:** ^1^ School of Science, STEM College RMIT University Bundoora Victoria Australia; ^2^ Mass Spectrometry Facility St Vincent's Institute of Medical Research Fitzroy Victoria Australia; ^3^ School of Biological Sciences Adelaide University Adelaide South Australia Australia; ^4^ St Vincent's Institute of Medical Research Fitzroy Victoria Australia; ^5^ Department of Medicine, St Vincent's Hospital University of Melbourne Fitzroy Victoria Australia

## Abstract

Immobilised Metal Affinity Chromatography (IMAC) is widely used to purify his‐tagged recombinant proteins from *Escherichia coli*. However, endogenous contaminants with histidine clusters, such as GFAT and PDH E1 proteins, are often co‐purified with the target protein. The low background strain LOBSTR‐RIL has been previously engineered with mutated forms of SlyD and ArnA that exhibit reduced binding to Ni^2+^ resin. In this study, the LOBSTR‐RIL strain was further modified to produce IMACulate(DE3), where we altered the *glmS* (encoding GFAT protein) and *aceE* (encoding PDH E1 protein) genes to reduce surface histidines. Proteins purified from this strain show reduced levels of GFAT contamination. No statistically significant difference was observed in the abundance levels of PDH E1 protein in the BL21(DE3)‐RIL, LOBSTR‐RIL and IMACulate(DE3) strains. The use of IMACulate(DE3) increases the purity of recombinant his‐tagged protein preparations with no additional effort or expense.

## 1 Introduction

A widely employed technique for purifying recombinant proteins from cell lysates utilises poly‐histidine affinity tags in combination with immobilised metal‐affinity chromatography (IMAC). This method leverages the interactions between a transition metal ion (most commonly Ni^2+^, occasionally Co^2+^ and rarely Zn^2+^) immobilised on a resin and clustered histidine residues, which interact strongly with these metal ion matrices. Peptides with sequences of consecutive histidine residues are effectively retained on IMAC column matrices (Bornhorst and Falke 2000). However, some host cell proteins possess a naturally high Ni^2+^ affinity through consecutive histidine residues and/or histidine patches formed from protein folding, which co‐purify along with the target protein contaminating the preparation. Host histidine‐rich protein contaminants from *Escherichia coli* expression have been identified by other groups (Bolanos‐Garcia and Davies 2006; Dobbs et al. 2020; Robichon et al. 2011).

Some of these co‐purifying, contaminant proteins are not required for optimal *E. coli* growth and can safely be eliminated by deletion of their coding sequence. Other host contaminant proteins however, are either essential or desirable for good growth kinetics. Andersen et al. (2013) has engineered a strain of BL21(DE3) *E. coli* with genomically modified versions of *slyD* and *arnA*, two common contaminants found from *E. coli* his‐tag Ni^2+^ and Co^2+^ IMAC purifications. Knocking out *arnA* or *slyD* in *E. coli* results in growth defects and is not practical in a protein production strain (Robichon et al. 2011; Roof et al. 1997). Thus, genomic modification was utilised to remove histidine residues, either by mutation or truncation, effectively reducing their affinity for Ni^2+^ and Co^2+^ and increasing the purity of his‐tagged proteins isolated by IMAC. Other host proteins identified following IMAC chromatography are the E1 subunit of the pyruvate dehydrogenase complex (PDH E1), encoded by the *aceE* gene, and L‐glutamine:D‐fructose‐6‐phosphate aminotransferase (GFAT), encoded by the *glmS* gene (Dobbs et al. 2020; Robichon et al. 2011). Pyruvate dehydrogenase complex catalyses the conversion of pyruvate to acetyl‐CoA (Moxley and Eiteman 2021), which can enter the citric acid cycle. L‐glutamine:d‐fructose‐6‐phosphate aminotransferase catalyses the first and rate‐limiting step of hexosamine biosynthesis. A product from this pathway UDP‐N‐acetylglucosamine is a key metabolite in the synthesis of peptidoglycan and lipopolysaccharides (Mouilleron et al. 2011). *E. coli* with *glmS* knocked out are non‐viable on LB media (Baba et al. 2006; Gerdes et al. 2003; Goodall et al. 2018), while *E. coli* with an *aceE* knockout can grow on LB media and M9 media supplemented with glycerol (Baba et al. 2006; Gerdes et al. 2003; Joyce et al. 2006). Even mild genetic alterations to the aceE gene, expressing PDH E1 proteins (with single amino acid mutations or in‐frame fusion proteins) can result in slower growth (Moxley et al. 2023; Robichon et al. 2011). Thus, neither gene are candidates for knockout in an *E. coli* protein expression strain. Instead, we strategically modified the *glmS* and *aceE* genes to reduce their histidine content with the aim to reduce binding to IMAC resins. Proteins purified from this derivative strain, IMACulate(DE3), showed significant purity improvements over LOBSTR and BL21(DE3) strains.

## 2 Materials and Methods

### 2.1 Designing Reduced Histidine Variants of GFAT and PDH E1 Proteins

The structure of the GFAT homodimer (PDB ID 1JXA) (Teplyakov et al. 2001), GFAT homo‐hexamer (PDB ID 3OOJ) (Mouilleron et al. 2012) and pyruvate dyhydrogenase E1 subunit protein (PDB ID 1L8A) (Arjunan et al. 2002) were analysed for clustered, surface‐exposed histidine residues. The degree of evolutionary conservation of each surface histidine was determined using the Conservation‐Colab (Graham 2023).

### 2.2 General Strains and Media

BL21(DE3) is *E. coli* strain B F^–^ *omp*T *gal dcm lon hsd*S_B_(r_B_–m_B_–) λ(DE3[*lacI lacUV5‐T7p07 ind*1 *Sam*7 *nin*5]) [malB^+^]_K‐12_. BL21(DE3) chemically competent cells were purchased from ThermoFisher Scientific. LOBSTR is a derivative of *E. coli* BL21(DE3) with modified *slyD* and *arnA* (henceforth referred to as *mslyD and marnA*) genes in the chromosome (Andersen et al. 2013). The protein product of the *marnA* gene has 4 amino acid substitutions (H359S, H361S, H592S and H593S). The protein product of the modified *mslyD* gene is a truncated form of the wildtype protein, removing 46‐residues from the C‐terminus. LOBSTR‐RIL is the LOBSTR strain harbouring the RIL plasmid (Agilent Technologies), which encodes for rare tRNAs that are not abundant in *E. coli*. LOBSTR‐RIL calcium chloride competent cells were purchased from Kerafast.

*E. coli* strains were routinely grown in Luria‐Bertani (LB) broth (10 g/L Tryptone, 5 g/L Yeast Extract, 10 g/L NaCl, pH 7) or 2 × YT broth (16 g/L Tryptone, 10 g/L Yeast Extract, 5 g/L NaCl, pH 7) with cultures incubated at the appropriate temperature (16°C, 30°C and 37°C). Media was supplemented with appropriate antibiotics at concentrations described in Table 1.

**Table 1:** Antibiotics used in this study.

Antibiotic	Concentration (µg/mL)	Purpose
Ampicillin	100	When strains harbour ampicillin expression vectors and recombineering vectors
Kanamycin	50	When harbouring plasmids with kanamycin resistance genes
	30	During recombineering steps with kanamycin resistance gene integrated into the bacterial chromosome
Chloramphenicol	20	When strains harbour the RIL plasmid

### 2.3 Plasmids

The pSIM6 plasmid (Datta et al. 2006) expresses λ red recombineering genes (*exo, bet, gam*) to allow genetic manipulations of *E. coli*. The pE_cre plasmid (Hao et al. 2021) constitutively expresses Cre recombinase from the P2 phage pE promoter. Both plasmids were selected for via their ampicillin resistance marker and have a temperature‐sensitive replicon which allows replication at 30°C and curing by culturing at 37°C.

Two proteins, transthyretin and kinesin family member 18 A (KIF18A) motor domain, were used to test the protein expression strains in this study, encoded by two expression plasmids. The pRSET_A_TTR has previously been described in Cody et al. (2022). pRSET_A_TTR has human transthyretin gene, encoding full length human TTR, under the control of the T7 promoter and an ampicillin resistance gene for selection. pET28a_KIF18A_MD carries the human kinesin family member 18A (KIF18A) motor domain, encoding KIF18A residues 1‐355, under the control of T7 promoter and a kanamycin resistance gene for selection.

### 2.4 DNA Construction

The targeting cassettes for recombineering procedures were created using a combination of DNA synthesis and overlap extension PCR. The gBlock sequences (IDT) are detailed in the Supporting Information. The primers used are summarised in Table S1.

**Figure 1:**
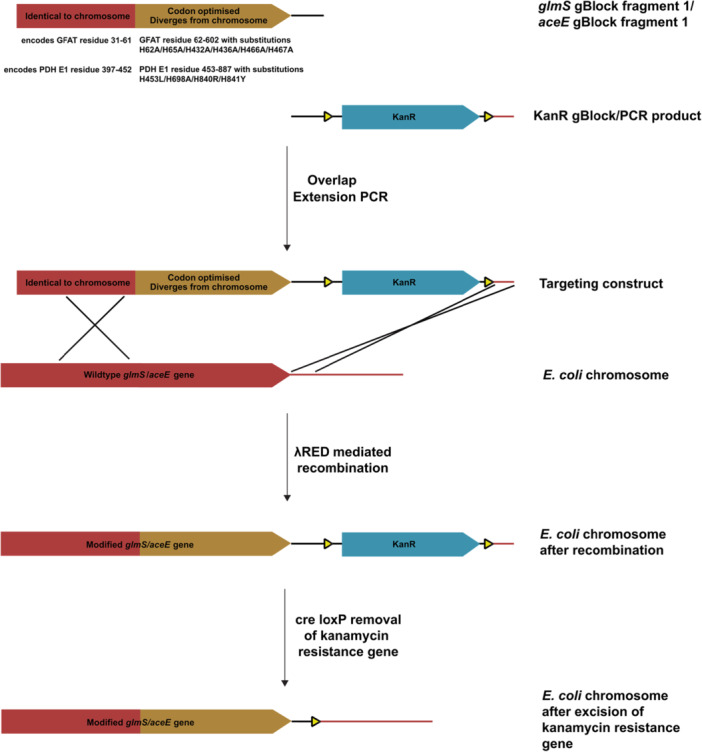
Recombineering strategy to mutate *glmS/aceE* gene.

The *glmS* targeting cassette was generated by joining two synthesised gBlock DNA fragments (IDT) using overlap extension‐PCR (Figure 1). The glmS gBlock contained the 108–184 bp region of the *glmS* open reading frame (ORF) (corresponding to residues 36–61 of the GFAT protein), fused in frame to a DNA sequence encoding residues 62–609 of GFAT, in which histidines at positions 62, 65, 432, 436, 466 and 467 were replaced with alanines. This construct was joined by a flanking sequence homologous to the antibiotic resistance gBlock. The sequence encoding residues 62–609 was codon‐optimised using the IDT codon optimisation tool (Shen and Packer 2022) to reduce similarity to the wildtype *glmS* gene, and minimise recombination at undesired areas. The kanR gBlock shared a flanking homologous sequence with the glmS gBlock, followed by the kanamycin resistance gene flanked by loxP LE and loxP RE sites (Albert et al. 1995). These two gBlocks were fused using OE‐PCR (Behle 2024) using primers glmS_6Ala_F and KanR_glmS_DS_R (Table S1).

The *aceE* targeting cassette was also generated by joining two DNA sequences using overlap extenstion PCR (Figure 1). The *aceE* gBlock contained the 1190–1356th bp sequence of the *aceE* ORF (corresponding to residues 397‐452 of the PDH E1 protein), sequence encoding residues 453–887 of PDH E1 with the histidine amino acid substitutions H453L, H698A, H840R, H841Y, followed by a flanking sequence to the kanR antibiotic resistance DNA fragment. The sequence encoding residues 62–609 was codon‐optimised using the IDT codon optimisation tool (Shen and Packer 2022) to reduce similarity to the wildtype *aceE* gene, to prevent undesired recombination. The second DNA fragment was made by amplifying the kanR gBlock with primers KanR_shortened_F and KanR_aceE_DS_R to introduce overlap with the aceE gBlock, followed by the kanamycin resistance gene flanked by loxP LE and loxP RE sites (Albert et al. 1995). The two DNA fragments were fused using overlap extension PCR (Behle 2024) using primers glmS_6Ala_F and KanR_glmS_DS_R.

### 2.5 Recombineering Procedure to Create IMACulate(DE3)

The genomic copy of *glmS* was modified in the following manner. pSIM6 was transformed into *E. coli* cells via electroporation and selected on LB agar supplemented with ampicillin (100 μg/mL) and chloramphenicol (20 μg/mL) at 30°C. The *glmS* targeting construct was transformed into the LOBSTR‐RIL pSIM6 via electroporation and plated LB agar supplemented with kanamycin (30 μg/mL) and chloramphenicol (20 μg/mL) at 37°C. Transformants with the intended recombination were screened via PCR using primers glmS_seq_F and glmS_seq_R (Table S1). These transformants were subsequently grown at 37°C overnight in 5 mL LB media to ensure plasmid curing of pSIM6, prior to being plated onto LB agar supplemented with chloramphenicol (20 μg/mL) at 37°C. The pE_Cre plasmid was transformed into the cured cells using TSS method (Chung et al. 1989) and selected on LB agar supplemented with ampicillin (100 μg/mL) and chloramphenicol (20 μg/mL) at 30°C. Transformants were subsequently grown at 37°C overnight in 5 mL LB media to ensure plasmid curing of pE_Cre. Successful excision of the kanamycin resistance marker was screened via PCR using primers glmS_seq_F and glmS_seq_R.

The genomic *aceE* gene was modified using the same recombineering strategy as *glmS*. The *aceE* targeting cassette was used for recombineering (Figure 1). Successful integration and subsequent antibiotic excision were confirmed via PCR using primers aceE_seq_F and aceE_seq_R. Genomic mutations of *glmS* and *aceE* were confirmed using Sanger sequence (Australian Genomics Research Facility). This strain carrying these mutations and the RIL plasmid was named IMACulate(DE3).

### 2.6 Growth Curves

All solid and liquid growth media during the growth rate measurement was supplemented with appropriate antibiotics (Table 1). BL21(DE3)‐RIL, LOBSTR‐RIL and IMACulate(DE3) carrying the same expression plasmid were streaked from glycerol stocks onto 2xYT agar and incubated overnight at 37°C. Single colonies were picked from agar and used to inoculate 100 µL of 2 × YT in 96 well microtitre plates (Corning Costar) and incubated overnight with shaking at 200 rpm. Overnight cultures were subcultured approximately 1:200 into 120 µL fresh 2 × YT media in 96 well microtitre plates. Optical density of the cultures at 600 nm (OD_600 nm_) throughout the experiment was monitored using an Omega plate reader (BMG Labtech). The subculture volumes were adjusted according to the overnight cultures to standardise the initial optical density of all cultures to OD_600 nm_ = 0.03. The cultures were incubated at 37°C for 2.5 h, after which 100 µL of cultures were induced with IPTG to a final concentration of 500 µM and incubated at the different induction temperatures (16°C, 25°C and 37°C) for an additional 19.5 h.

### 2.7 Protein Expression in *E. coli* and IMAC Purification Test

BL21(DE3)‐RIL, LOBSTR‐RIL and IMACulate(DE3) carrying the same expression plasmids were streaked from glycerol stocks as per the growth curve experiments. A single colony was picked from each agar plate to inoculate 5 mL of 2 × YT supplemented with antibiotics (Table 1) and incubated overnight at 37°C. Overnight cultures were used to inoculate 1 L of 2 × YT broth and grown until OD_600 nm_ = 0.5–0.7 and subsequently induced with IPTG to a final concentration of 100 µM. The cultures were incubated at 16°C overnight. Cells were harvested by centrifugation at 3900 × *g* for 10 min, prior to resuspension in 10 mL of buffer A (20 mM Tris‐HCl, 100 mM KCl, 40 mM imidazole, 5 mM MgCl_2_, 1 mM BME, pH 7.5). The resuspended cells were lysed by sonication (Branson Digital Sonifier 250 Cell Disruptor; 2 min at 40% amplitude; 1 s pulse with 3 s rest). Lysates were clarified by centrifugation at 3900 × *g* for 30 min, prior to filtering through 0.22 µM syringe filters (Sartorius). The soluble fraction was incubated with loose Ni^2+^ charged resin (Genscript) for 30 min at 4°C with shaking agitation. The resin was washed five times with 1.5 mL of buffer A and protein was eluted with 100 µL of buffer B (20 mM Tris‐HCl, 100 mM KCl, 500 mM imidazole, 5 mM MgCl_2_, 1 mM BME, pH 7.5). Eluted protein was analysed on 12% Bolt Bis‐Tris Plus mini‐gels (ThermoFisher Scientific) or 4‐15% Mini‐Protean TGX gels (BioRad) and stained with InstantBlue Coomassie Protein Stain (Abcam).

### 2.8 Mass Spectrometry Analysis of Purified Protein

Aliquots with 10 μg of each purified protein were initially reduced with 10 mM dithiothreitol (DTT) at 55°C for 30 min, then alkylated with 55 mM iodoacetamide (IAA) for 30 min at room temperature in the dark. The digestion was performed by adding trypsin at a 1:50 enzyme‐to‐substrate ratio (w/w) and incubating at 37°C overnight. The reaction was quenched by adding formic acid to a final concentration of 0.5%. Peptides were desalted through C18 Stage‐Tips, then vacuum dried prior to analysis.

Peptide mixtures were separated using an Ultimate 3000 nanoRSLC system (Dionex, CA, US) coupled to an Orbitrap Fusion Lumos mass spectrometer (Thermo Fisher Scientific, CA, US). Samples were first trapped in a PepMap Neo Trap Cartridge (Thermo Fisher Scientific), then loaded onto a Double nanoViper PepMap Neo UHPLC Column (75 μM × 50 cm, 2 μM particle size, Thermo Fisher Scientific) maintained at 40°C. The following solvents were employed: Solvent A was 0.1% formic acid in water, and Solvent B was 0.1% formic acid in 80% acetonitrile. Peptides were eluted with a linear gradient from 5% to 35% Solvent B over 30 min, increased to 45% over 5 min, ramped to 90% over 5 min, and held for an additional 15 min. The flow rate was set to 230 nL/min. 1 μg of peptide was injected for each run.

The Orbitrap Fusion Lumos was operated in data‐dependent acquisition mode. Survey full MS scans were acquired at a resolution of 120,000 (at m/z 200) over a mass range of m/z 350–1500 with the 20 most abundant ions selected for higher‐energy collision dissociation (HCD) fragmentation. MS/MS spectra were acquired at a resolution of 30,000 with a dynamic exclusion duration of 45 s. The normalised collision energy was set to 30%. Automatic gain control targets were set at 4e5 for MS1 and 5e4 for MS2. Maximum injection times were 50 ms for MS1 and 100 ms for MS2.

Raw LC‐MS/MS data were processed using Proteome Discoverer 2.5 software (Thermo Fisher Scientific). Peptide identification was performed using Sequest HT against the *E. coli* proteome database as well as collection of all plasmid‐expressed protein sequences. Trypsin was specified as the enzyme with up to two missed cleavages allowed. Carbamidomethylation of cysteine was set as a fixed modification, with methionine oxidation and protein N‐terminal acetylation included as variable modifications. A 1% false discovery rate (FDR) threshold was applied at both peptide and protein levels. Protein abundances were quantified by summing the intensities of top 3 abundant and shared peptides assigned to each targeted protein.

## 3 Results

L‐glutamine:d‐fructose‐6‐phosphate aminotransferase (GFAT; *glmS*) and E1 subunit of the pyruvate dehydrogenase complex (PDH E1; *arnA*) both contain surface‐exposed histidine clusters

**Figure 2:**
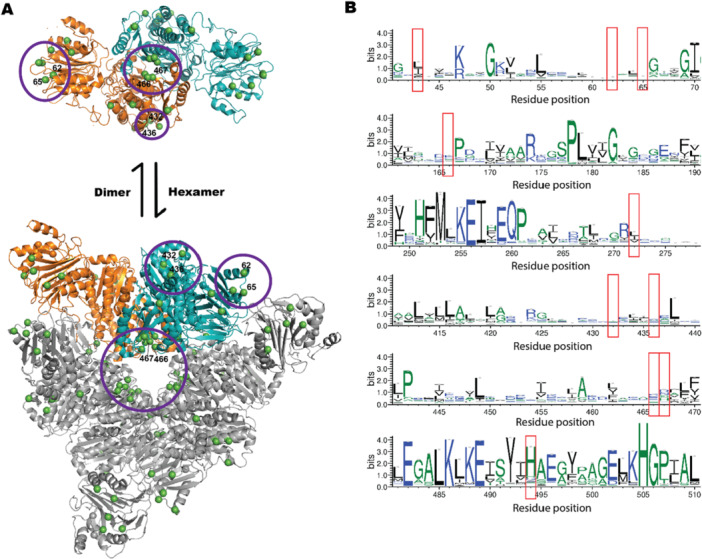
GFAT exists in equilibrium dimeric forms (PDB ID 1JXA) and hexameric forms (PDB ID 3OOJ)(Mouilleron et al. 2012). Surface histidine residues are coloured green. Histidine patches are highlighted with purple ellipses. Surface exposed histidine residues are shown as green spheres. Histidine residues that were mutated for IMACulate(DE3) are indicated with their residue number. (B) Logo plot of Conservation‐colab evolutionary analysis of histidine residues found in the three largest surface clusters on GFAT (Graham 2023). Red boxes indicate positions with surface‐exposed histidines. Sections of the logo plot have been omitted to emphasise residue positions from major histidine patches.

GFAT exists in an equilibrium between two structural forms: an active dimer and an inactive hexamer, which is a trimer of dimers (Mouilleron et al. 2012). Surface exposed histidine residues were considered in both forms (Figure 2a). Three main histidine clusters were observed. H466, H467, H494 formed a tight cluster of 6 histidines across the monomer‐monomer interface in both the dimer and hexameric structures. In the hexamer, these clusters are all situated within a central cavity, creating a channel containing 18 accessible histidines. We postulated that evolutionary conserved histidines are likely to be functionally important for GFAT and PDH E1, while less conserved histidines would be good targets for mutagenesis. Evolutionary analysis using Conservation‐colab was used to identify the evolutionary conservation of each histidine in homologous structures (Figure 2b). Briefly, this script uses MMSeq. 2 to identify sequences related to the target protein (Steinegger and Söding 2017) and uses a multiple sequence alignment identify to the degree of conservation in each amino acid position. Of the histidines in the central channel cluster, residue 494 showed a high conservation, while residues 466 and 467 were significantly more variable and therefore was not mutated. Within each monomer in both dimeric and hexameric forms, there are two histidine surface clusters. One cluster is formed by H43, H62, H65 and H166. From this cluster, positions 62 and 65 showed high variability, with the other positions showing less residue variability. The second cluster is formed by H274, H432 and H436. Positions 432 and 436 showed high variability, whilst position 274 showed less variability. Thus, to target all three histidine patches, the less conserved residue positions 62, 65, 432, 436, 466 and 467 were selected for mutation. Robichon et al. (2011) previously proposed to reduce GFAT binding to Ni^2+^ resin using the same six histidines and inserting alanine mutations (H62A, H65A, H432A, H436A, H466A and H467A) to disrupt the three main histidine patches identified in our analyses. Thus, we adopted the same mutations for our low background strain. However, differing from their approach, these mutations were introduced into a pre‐existing low background strain (LOBSTR‐RIL) with a codon optimised targeting cassette for simplified recombineering.

**Figure 3:**
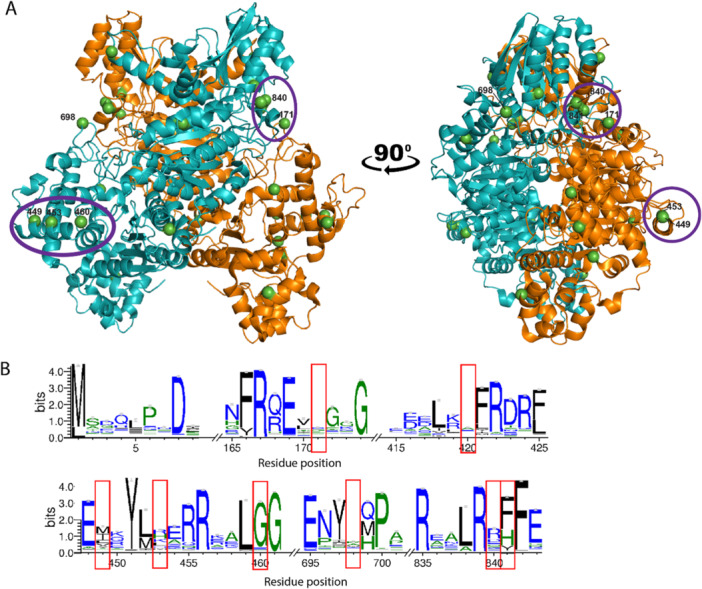
Surface histidines are present on PDH E1 (A) The two largest histidine patches on the crystal structure of PDH E1 dimer are highlighted with purple ellipses (PDB ID 1L8A). Surface exposed histidine residues are shown as green sticks. One cluster consists of H449, H453 and H460. The other cluster consists of H840, H841 and H171 contributed from the other monomer. Histidine residues of PDH E1 that were mutated for IMACulate(DE3) are indicated with their residue number. (B) Logo plot of Conservation‐colab evolutionary analysis of histidine residues found in the two largest surface clusters on PDH E1 (16). Red boxes indicate positions with surface‐exposed histidines. Sections of the logo plot have been omitted to emphasise residue positions from major histidine patches.

PDH E1 complex is found as dimers within *E. coli* (Danson et al. 1978), and is a protein essential for growth of *E. coli* (Arjunan et al. 2002). The crystal structure of dimeric PDH E1 (PDB ID 1L8A) was analysed for surface‐histidine clusters, with two largest histidine clusters highlighted in Figure 3A. One cluster is composed of H449, H453 and H460. The other cluster is composed on H840, H841 and H171 from the other monomer. Each cluster is observed twice, once on each monomer. Conservation‐colab (Graham 2023) was used to perform evolutionary analysis of residue conservation in these positions and to determine other amino acids commonly found in these positions Figure 3B. Histidine substitutions H453L, H840R and H841Y were selected. These residues were variable at those positions and were mutated to the amino acids commonly found in these positions to reduce the probability of deleterious mutations. The mutation H698A is not part of the two large clusters but was also mutated in IMACulate(DE3), as it is a highly variable position across similar proteins, to further reduce surface histidine abundance and lower risk of being a deleterious mutation.

### 3.1 IMACulate(DE3) Displays No Significant Growth Differences From Its Parental Strain LOBSTR‐RIL

**Figure 4:**
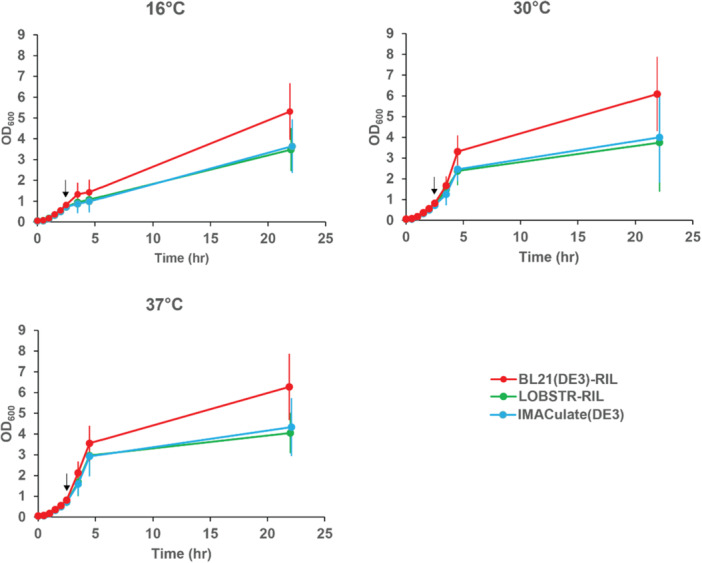
LOBSTR‐RIL and IMACulate(DE3) show similar growth rates in liquid LB media across different induction conditions. Both strains are observed to have a reduction in final culture densities compared to BL21(DE3)‐RIL. The optical density at 600 nm of the cultures were measured after initial synchronisation at time = 0 h. The cultures were grown at 37°C until OD_600_ = 0.6–0.8 (indicated by arrow), then protein expression was induced with IPTG added to a final concentration of 500 µM and cultures incubated at 16°C, 25°C or 37°C. Error bars correspond to 95% confidence interval (*n* = 3).

The designed mutations (10 amino acid changes in total) were made according to the strategy in Figure 1 and confirmed by sequencing. The modified *glmS* gene (*mglmS*) was introduced first, prior to introducing the modified *aceE* gene (*maceE*). The growth of BL21(DE3)‐RIL, LOBSTR‐RIL and IMACulate(DE3) was examined in liquid culture to determine if the modified *mglmS* and *maceE* genes had any effect on growth rate (Figure 4). The modifications in IMACulate(DE3) did not have an observable effect on the growth of the strains, compared to its parental LOBSTR‐RIL strain. No significant differences were observed in the growth rate pre‐ and post‐induction between the strains and the final OD_600_ after overnight induction were very similar. However, BL21(DE3)‐RIL were observed to reach slightly higher optical densities compared to LOBSTR‐RIL and IMACulate(DE3), with the effect most notable at 16°C.

### 3.2 IMAC Purifications Using IMACulate(DE3) Have Fewer Contaminating Bands Than BL21(DE3) and LOBSTR‐RIL

**Figure 5:**
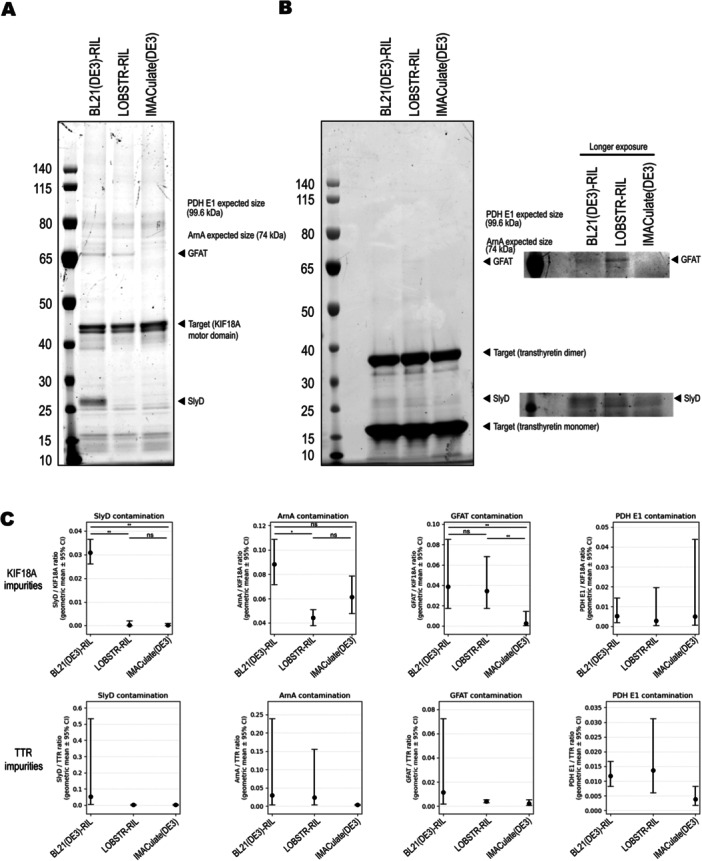
Ni^2+^ IMAC purifications of KIF18A motor domain (A) and transthyretin (B) from IMACulate(DE3) show reduced GFAT impurity relative to BL21(DE3)‐RIL and LOBSTR‐RIL. Protein eluted from Ni^2+^ resin was run on 4%–12% BisTris SDS‐PAGE gel (Thermo Fisher Scientific) using 1 x MOPS buffer. Gels were stained for 15 min using InstantBlue Commassie dye (Abcam) and destained in deionised water overnight. Gels were imaged on a Chemidoc (BioRad) using Epi‐Red setting for enhanced signal to noise. As expected, the SlyD impurity was also observed to be reduced in the KIF18A motor domain purification and slightly reduced in TTR purification for IMACulate(DE3) and LOBSTR‐RIL relative to BL21(DE3)‐RIL. (C) Normalised impurity abundances detected using label‐free mass spectrometry, plotted as geometric means ± 95% CI (*n* = 3). Impurity abundances were normalised against the abundance of target protein. Abundances of target proteins and impurities were calculated as the sum of the top 3 abundant and shared peptides of the protein of interest across the samples. Contaminant abundances with statistically significant differences were determined using one‐way ANOVA. Post‐hoc Tukey HSD analysis was performed to identify strains that are statistically different. ns indicates *p* > 0.05, * indicates *p* < 0.05, ** indicates *p* < 0.01.

The protein purified from BL21(DE3)‐RIL, LOBSTR‐RIL and IMACulate(DE3) were analysed by SDS‐PAGE (Figure 5). Two expression plasmids were tested, the motor domain of human KIF18A (KIF18A_MD) in a pET28a plasmid (Figure 5A), and human transthyretin (TTR) in the pRSET_A plasmid (Figure 5B). These represent low expression and high expression examples, respectively. From previous experience (unpublished data), KIF18A_MD exhibits low protein expression in *E. coli* and obtaining > 85% pure protein from Ni^2+^ IMAC requires extensive washing with high imidazole (~200 mM) wash buffers. TTR overexpresses in *E. coli* very well and can be purified to high purity in large quantities.

A band of 66.8 kDa observed in the BL21(DE3)‐RIL and LOBSTR‐RIL purifications for both test proteins, was greatly reduced in both IMACulate purifications. This effect was consistently observed for three repeated purifications (Figure 5A,B and Figure S2). This mass corresponds to the expected size of GFAT. These improvements were also similarly reflected in mass spectrometry quantification for KIF18A purifications, with an observed 15‐fold reduction with GFAT levels between LOBSTR‐RIL and IMACulate(DE3). GFAT levels in TTR purifications from IMACulate(DE3) were on average 45% lower than LOBSTR‐RIL. However, this difference was not statistically significant (*p* = 0.19), likely due to high variability in mass spectrometry quantification. (Figure 5C). LOBSTR‐RIL was originally engineered to eliminate the ArnA and SlyD impurities (Andersen et al. 2013). The SlyD impurities were observed to be greatly reduced in both the LOBSTR‐RIL and its derivative created here IMACulate(DE3) compared to BL21(DE3)‐RIL, for the KIF18A_MD purification and slightly reduced for TTR purification in SDS‐PAGE. This reduction was statistically significant when quantified using MS for KIF18A purifications, but not for TTR purifications. This results highlights the importance of low contaminant expression strains like IMACulate(DE3) for low expression targets like KIF18A.

Similar improvements in purity were independently reproduced with KIF18A motor domain and transthyretin expressing *E. coli* cultures in altered expression and purification conditions. These were induced with higher IPTG levels (500 µM) and resin less stringently washed during purification (two 1.5 mL washes instead of five 1.5 mL washes) to allow more impurities to be retained (Figure S3). In this less stringent purification, reduced GFAT impurities was observed in IMACulate(DE3) for both purifications. Reduced levels of ArnA and SlyD impurities were observed in both IMACulate(DE3) and LOBSTR‐RIL on SDS‐PAGE, as expected.

The other impurity mutated in this study was *aceE* (PDH E1), with an expected size of 99.7 kDa. The low impurity levels in the transthyretin purifications and the presence of similar sized contaminants in the case KIF18A motor domain purifications made it difficult to identify the PDH E1 impurity via SDS‐PAGE. Mass spectrometry analysis showed no statistically significant differences in PDH E1 abundance among the three strains across both test protein purifications. However, based on SDS‐PAGE analysis, PDH E1 is a minor impurity, when compared to SlyD and GFAT, that is not likely to significantly change the purity of the protein.

## 4 Discussion

The *E. coli* strain BL21(DE3) is one of the most widely employed workhorses for the production of recombinant proteins (Yoon et al. 2009). It was created through the integration of the DE3 prophage into the λ attachment site, allowing T7 RNA polymerase to be used to drive protein expression from the T7 promoter present on the expression plasmid (Studier and Moffatt 1986). IMACulate(DE3) represents an optimised derivative of BL21(DE3) for the production of His‐tagged proteins by IMAC purification, enabling higher protein purity to be achieved with no additional reagents or purification steps.

Generation of IMACulate(DE3) by recombineering involved multiple rounds of kanamycin and ampicillin selection, followed by removal of the corresponding resistance genes in the final strain. The two expression plasmids tested here, a pRSET_A and a pET28a plasmid, required ampicillin and kanamycin for selection, respectively, demonstrating that kanamycin & ampicillin selection are compatible with IMACulate(DE3).

Although, the average measured abundance of impurities of SlyD, ArnA, GFAT and PDH E1 in TTR purifications in IMACulate(DE3) were lower than both LOBSTR‐RIL and BL21(DE3)‐RIL (Figure 5 C), our analysis failed to identify any statistically significant differences between the strains with α = 0.05 cutoff. This is likely due to the large quantitative variability in the abundance measurements by mass spectrometry. One contributing factor for this effect may be the much lower absolute levels of impurities in these purifications compared to KIF18A, leading to more noise in the measurements. However, the qualitative loss of the GFAT and SlyD bands consistently across three independent purifications of TTR demonstrates the improved purity of protein preparations. In a high yield protein like TTR, the hexahistidine tag likely out‐competes the impurities in binding to the IMAC resin. The benefit of our strain is better observed in the purification of proteins that express less well (such as KIF18A) where impurities are more prominent, and additional purification steps may yield insufficient protein for downstream applications.

Although the protein preparations from IMACulate(DE3) showed increased purity compared to both BL21(DE3)‐RIL and LOBSTR‐RIL (Figure 5), our results were unable to specifically determine the exact contributors to this increased purity. Our approach changes the codons in the open reading frame of the genes, which could in principle alter their expression levels in the cell. The GFAT_6Ala_ protein was previously demonstrated to display significantly lower affinity to Ni^2+^ resin (Robichon et al. 2011) and was observed to have reduced levels in our protein purifications. In contrast, no clear reduction in PDH E1 levels was observed, and the effect of the introduced mutations on its affinity for Ni²⁺ resin remains unclear. However, as PDH E1 was a minor impurity under our conditions and the mutations did not impact growth rate or significantly reduce protein purity, it was kept in the final strain. The GFAT_6Ala_ was previously demonstrated to be active by Robichon et al. (2011), as complementation assays with GFAT_6Ala_ able to restore the cell viability of *E. coli* with a loss‐of‐function modification of GFAT, in the absence of glucosamine and N‐acetylglucosamine. The mutant PDH E1 protein was not functionally characterised in this study. It plays a central role in bioenergetics, bridging glycolysis and the citric acid cycle (Moxley and Eiteman 2021). *E. coli* unable to produce functional pyruvate dehydrogenase complexes cannot produce acetyl‐CoA from pyruvate and require additional carbon sources such as acetate (Langley and Guest 1978). 2xYT media, a rich but undefined media, was used in this study. Thus, the carbon sources within the media were not strictly controlled for. We also cannot rule out changes in protein activity/protein levels that have been offset by compensatory changes in the cell. However, the similar growth rates, final culture densities, and similarities in protein expression levels obtained between IMACulate(DE3) and LOBSTR‐RIL suggests the mutations have not significantly altered metabolism, at least in 2xYT media. A previous attempt at modifying PDH E1 in BL21(DE3) derivatives, resulted in reduced growth without acetate dependence (Robichon et al. 2011). Our careful selection of less‐conserved histidines through evolutionary analysis and substitutions to residues frequently in these positions likely avoided any detrimental effects to PDH E1 activity.

IMACulate(DE3) and LOBSTR‐RIL achieved lower culture densities relative to BL21(DE3)‐RIL. Andersen et al. (2013) tested the parental strains without the RIL plasmids and showed there is no significant difference in growth rates and final densities in BL21(DE3) and LOBSTR. All our tested strains possessed the RIL‐plasmid. Although unlikely, we cannot rule out that the RIL plasmids changed the growth rate and final densities between BL21(DE3)‐RIL and LOBSTR‐RIL. LOBSTR‐RIL was obtained from Kerafast. BL21(DE3) was obtained from Thermofisher Scientific and transformed with RIL‐plasmid. It is more likely that potential handling of bacteria by either manufacturer has caused heritable epigenetic or genetic changes over time in the either strain, resulting in deviations in growth rate. We demonstrate in our study that our genetic manipulation of LOBSTR‐RIL has not created any additional growth changes.

The approach used in this study also allows for additional incremental improvements of IMACulate(DE3) in the future. The use of codon optimised targeting cassettes during recombination simplifies the recombination procedures by limiting λ‐RED mediated recombination to only desired regions. The changes in the ORF do not create observable growth differences in the final strain. The use of loxP LE and loxP RE sites in antibiotic excision generates a loxP DNA scar that also resist further Cre‐mediated excisional recombination (19, 22). Modifications to the genes responsible for protein impurities could be tackled with the same approach with a lower risk of large unintended recombination events from previous loxP scars. Our approach represents an improvement to the previous approach of engineering LOBSTR using FRT sites that can result in genomic instability if further engineering is performed using FLP‐mediated FRT recombination.

There remains impurities observed in our purifications with IMACulate(DE3), with much of these impurities observed in the 10‐25 kDa range. MS analysis of these samples identified enriched contaminants including cAMP receptor protein (23.6 kDa), Hfq (11.2 kDa) and ferric uptake regulator protein (16.8 kDa). These proteins have been independently identified as common *E. coli* IMAC contaminants (Bolanos‐Garcia and Davies 2006; Robichon et al. 2011), and could be good candidates for additional strain engineering to further improve these low contaminant strains.

While we show utility of the IMACulate(DE3) expression system for both high and low yield protein targets, we especially recommend IMACulate(DE3) for significant contaminant reductions in low expressing proteins. Researchers interested in accessing the strain can contact the corresponding author.

## Author Contributions

**Jia Q. Truong:** conceptualisation, methodology, experiments – strain engineering and protein purification, data analysis, writing – original draft, review and editing. **Dingyi Yu:** experiments – mass spectrometry, data analysis, writing – original draft, editing. **Nan Hao:** methodology, experimental materials, writing – original draft, review and editing. **Chris Langendorf:** methodology, provided mass spectrometry resources, data analysis, writing – original draft, review and editing. **Keith. E. Shearwin:** methodology, writing – original draft, review and editing. **Jessica K. Holien:** methodology, writing – original draft, review and editing.

## Conflicts of Interest

The authors declare no conflicts of interest.

## Supporting information

Supporting File

## Data Availability

The data that supports the findings of this study are available in the supporting material of this article.

## References

[bit70244-bib-0001] Albert, H. , E. C. Dale , E. Lee , and D. W. Ow . 1995. “Site‐Specific Integration of DNA Into Wild‐Type and Mutant Lox Sites Placed in the Plant Genome.” Plant Journal 7, no. 4: 649–659. 10.1046/j.1365-313X.1995.7040649.x. 7742860

[bit70244-bib-0002] Andersen, K. R. , N. C. Leksa , and T. U. Schwartz . 2013. “Optimized E. coli Expression Strain Lobstr Eliminates Common Contaminants From His‐Tag Purification.” Proteins: Structure, Function, and Bioinformatics 81, no. 11: 1857–1861. 10.1002/prot.24364. PMC4086167 23852738

[bit70244-bib-0003] Arjunan, P. , N. Nemeria , A. Brunskill , et al. 2002. “Structure of the Pyruvate Dehydrogenase Multienzyme Complex E1 Component From Escherichia coli at 1.85 Å Resolution.” Biochemistry 41, no. 16: 5213–5221. 10.1021/bi0118557. 11955070

[bit70244-bib-0004] Baba, T. , T. Ara , M. Hasegawa , et al. 2006. “Construction of Escherichia coli K12 In‐Frame, Single‐Gene Knockout Mutants: The Keio Collection.” Molecular Systems Biology 2, no. 1: 2006.0008. 10.1038/msb4100050. PMC1681482 16738554

[bit70244-bib-0005] Behle, A. 2024. *Overlap extension PCR*. Protocols.IO. https://www.protocols.io/view/overlap-extension-pcr-x54v9xkzv3eq/v1.

[bit70244-bib-0006] Bolanos‐Garcia, V. M. , and O. R. Davies . 2006. “Structural Analysis and Classification of Native Proteins From E. coli Commonly Co‐Purified by Immobilised Metal Affinity Chromatography.” Biochimica et Biophysica Acta (BBA) ‐ General Subjects 1760, no. 9: 1304–1313. 10.1016/j.bbagen.2006.03.027. 16814929

[bit70244-bib-0007] Bornhorst, J. A. , and J. J. Falke . 2000. “Purification of Proteins Using Polyhistidine Affinity Tags.” Methods in Enzymology 326: 245–254. 10.1016/s0076-6879(00)26058-8. 11036646 PMC2909483

[bit70244-bib-0008] Chung, C. T. , S. L. Niemela , and R. H. Miller . 1989. “One‐Step Preparation of Competent Escherichia coli: Transformation and Storage of Bacterial Cells in the Same Solution.” Proceedings of the National Academy of Sciences 86, no. 7: 2172–2175. 10.1073/pnas.86.7.2172. PMC286873 2648393

[bit70244-bib-0009] Cody, V. , J. Q. Truong , B. A. Holdsworth , et al. 2022. “Structural Analysis of the Complex of Human Transthyretin With 3′,5′‐Dichlorophenylanthranilic Acid at 1.5 Å Resolution.” Molecules 27, no. 21: 7206. 10.3390/molecules27217206. 36364032 PMC9659241

[bit70244-bib-0010] Danson, M. J. D. , E. A. Hooper , and R. N. Perham . 1978. “Intramolecular Coupling of Active Sites in the Pyruvate Dehydrogenase Multienzyme Complex of Escherichia coli.” Biochemical Journal 175, no. 1: 193–198. 10.1042/bj1750193. 367364 PMC1186054

[bit70244-bib-0011] Datta, S. , N. Costantino , and D. L. Court . 2006. “A Set of Recombineering Plasmids for Gram‐Negative Bacteria.” Gene 379: 109–115. 10.1016/j.gene.2006.04.018. 16750601

[bit70244-bib-0012] Dobbs, J. M. , M. L. Jenkins , and J. E. Burke . 2020. “Escherichia coli and Sf9 Contaminant Databases to Increase Efficiency of Tandem Mass Spectrometry Peptide Identification in Structural Mass Spectrometry Experiments.” Journal of the American Society for Mass Spectrometry 31, no. 10: 2202–2209. 10.1021/jasms.0c00283. 32869988

[bit70244-bib-0013] Gerdes, S. Y. , M. D. Scholle , J. W. Campbell , et al. 2003. “Experimental Determination and System Level Analysis of Essential Genes in Escherichia coli Mg1655.” Journal of Bacteriology 185, no. 19: 5673–5684. 10.1128/jb.185.19.5673-5684.2003. 13129938 PMC193955

[bit70244-bib-0014] Goodall, E. , A. Robinson , I. G. Johnston , et al. 2018. “The Essential Genome of Escherichia coli K‐12.” mBio 9, no. 1: e02096‐17. 10.1128/mbio.02096-17. 29463657 PMC5821084

[bit70244-bib-0015] Graham, C. 2023. *Conservation‐Colab: Conservation to 3D structure Colab v1.0.1*.

[bit70244-bib-0016] Hao, N. , Q. Chen , I. B. Dodd , and K. E. Shearwin . 2021. “The pIT5 Plasmid Series, an Improved Toolkit for Repeated Genome Integration in E. coli.” ACS Synthetic Biology 10, no. 7: 1633–1639. 10.1021/acssynbio.1c00215. 34190535

[bit70244-bib-0017] Joyce, A. R. , J. L. Reed , A. White , et al. 2006. “Experimental and Computational Assessment of Conditionally Essential Genes in Escherichia coli.” Journal of Bacteriology 188, no. 23: 8259–8271. 10.1128/jb.00740-06. 17012394 PMC1698209

[bit70244-bib-0018] Langley, D. , and J. R. Guest . 1978. “Biochemical Genetics of the ‐Keto Acid Dehydrogenase Complexes of Escherichia coli K 12: Genetic Characterization and Regulatory Properties of Deletion Mutants.” Journal of General Microbiology 106, no. May: 103–117. 10.1099/00221287-106-1-103. 349114

[bit70244-bib-0019] Mouilleron, S. , M. A. Badet‐Denisot , B. Badet , and B. Golinelli‐Pimpaneau . 2011. “Dynamics of glucosamine‐6‐phosphate Synthase Catalysis.” Archives of Biochemistry and Biophysics 505, no. 1: 1–12. 10.1016/j.abb.2010.08.008. 20709015

[bit70244-bib-0020] Mouilleron, S. , M.‐A. Badet‐Denisot , L. Pecqueur , et al. 2012. “Structural Basis for Morpheein‐Type Allosteric Regulation of Escherichia coli Glucosamine‐6‐phosphate Synthase.” Journal of Biological Chemistry 287, no. 41: 34533–34546. 10.1074/jbc.M112.380378. 22851174 PMC3464560

[bit70244-bib-0021] Moxley, W. C. , R. E. Brown , and M. A. Eiteman . 2023. “Escherichia coli Acee Variants Coding Pyruvate Dehydrogenase Improve the Generation of Pyruvate‐Derived Acetoin.” Engineering in Life Sciences 23, no. 3: e2200054. 10.1002/elsc.202200054. 36874610 PMC9978916

[bit70244-bib-0022] Moxley, W. C. , and M. A. Eiteman . 2021. “Pyruvate Production by Escherichia coli by Use of Pyruvate Dehydrogenase Variants.” Applied and Environmental Microbiology 87, no. 13: e0048721. 10.1128/AEM.00487-21. 33863707 PMC8315933

[bit70244-bib-0023] Robichon, C. , J. Luo , T. B. Causey , J. S. Benner , and J. C. Samuelson . 2011. “Engineering Escherichia coli BL21(DE3) Derivative Strains to Minimize E. coli Protein Contamination After Purification by Immobilized Metal Affinity Chromatography.” Applied and Environmental Microbiology 77, no. 13: 4634–4646. 10.1128/AEM.00119-11. 21602383 PMC3127686

[bit70244-bib-0024] Roof, W. D. , H. Q. Fang , K. D. Young , J. Sun , and R. Young . 1997. “Mutational Analysis of Slyd, An Escherichia coli Gene Encoding a Protein of the FKBP Immunophilin Family.” Molecular Microbiology 25, no. 6: 1031–1046. 10.1046/j.1365-2958.1997.5201884.x. 9350861

[bit70244-bib-0025] Shen, S. , and H. Packer (2022). *Codon Optimization Tool Makes Synthetic Gene Design Easy*. https://sg.idtdna.com/pages/tools/codon-optimization-tool.

[bit70244-bib-0026] Steinegger, M. , and J. Söding . 2017. “MMseqs. 2 Enables Sensitive Protein Sequence Searching for the Analysis of Massive Data Sets.” Nature Biotechnology 35, no. 11: 1026–1028. 10.1038/nbt.3988. 29035372

[bit70244-bib-0027] Studier, F. W. , and B. A. Moffatt . 1986. “Use of Bacteriophage T7 RNA Polymerase to Direct Selective High‐Level Expression of Cloned Genes.” Journal of Molecular Biology 189, no. 1: 113–130. 10.1016/0022-2836(86)90385-2. 3537305

[bit70244-bib-0028] Teplyakov, A. , G. Obmolova , B. Badet , and M.‐A. Badet‐Denisot . 2001. “Channeling of Ammonia in Glucosamine‐6‐Phosphate Synthase.” Journal of Molecular Biology 313, no. 5: 1093–1102. 10.1006/jmbi.2001.5094. 11700065

[bit70244-bib-0029] Yoon, S. H. , H. Jeong , S. K. Kwon , and J. F. Kim . 2009. “Genomics, Biological Features, and Biotechnological Applications of B: “Is B for Better?” Systems Biology and Biotechnology of Escherichia coli 1, 1–17. 10.1007/978-1-4020-9394-4_1.

