# Supplementary material for: IMACulate(DE3), an E. coli Strain for High Purity His‐Tagged Protein Purifications
Source: Biotechnol Bioeng. 2026 May 19;123(8):2127–38. doi: 10.1002/bit.70244 (PMC13397214; doi:10.1002/bit.70244)
Supplement: Supplementary file 1 — Supporting File [file BIT-123-2127-s001.docx]

# Supplementary information

glmS gBlock sequence

TGTTGATGCGGAAGGTCATATGACCCGCCTGCGTCGCCTCGGTAAAGTCCAGATGCTGGCTCAGGCAGCGGAAGAAGCTCCACTTGCTGGTGGCACCGGGATTGCTCACACACGGTGGGCCACTCACGGCGAGCCATCAGAGGTGAATGCTCATCCTCATGTATCGGAACACATTGTGGTCGTCCACAACGGTATCATTGAAAATCACGAGCCTCTCCGGGAAGAGCTCAAGGCACGTGGCTACACTTTTGTAAGTGAAACTGATACAGAAGTAATCGCACATCTCGTAAATTGGGAACTGAAGCAAGGTGGGACCTTACGGGAGGCGGTCTTACGCGCAATCCCTCAGTTGCGGGGCGCCTACGGCACCGTGATCATGGACTCGCGGCACCCAGATACGTTATTAGCTGCGCGTTCCGGGAGTCCACTGGTTATCGGGCTGGGGATGGGGGAGAACTTCATTGCCTCCGACCAGCTGGCGTTACTCCCGGTGACCCGTCGTTTTATTTTCCTTGAGGAAGGCGACATTGCGGAGATTACACGGCGCTCTGTAAATATCTTTGATAAAACAGGCGCAGAGGTCAAACGGCAGGACATTGAAAGCAATTTGCAATACGATGCCGGTGATAAAGGTATTTATCGCCATTATATGCAGAAAGAAATTTATGAGCAACCTAATGCGATCAAGAATACACTGACCGGGCGGATCTCACACGGCCAAGTCGATTTGTCAGAACTCGGTCCAAATGCGGACGAGCTTTTAAGTAAGGTGGAGCACATCCAGATCTTAGCTTGCGGTACCAGTTATAATTCGGGCATGGTGAGTCGGTATTGGTTCGAATCTCTCGCGGGGATCCCTTGCGACGTTGAAATCGCCTCAGAATTCCGGTACCGTAAGTCTGCAGTGCGCCGCAACAGTCTCATGATCACGCTCAGCCAATCAGGCGAAACAGCAGATACTCTGGCCGGGCTGCGTCTTAGTAAGGAACTCGGCTACTTAGGGAGTTTAGCCATCTGCAATGTGCCAGGGTCATCGTTAGTTCGGGAATCTGACCTGGCGCTTATGACCAATGCGGGTACAGAAATCGGTGTGGCGAGCACTAAAGCATTCACCACACAGTTGACGGTCCTCCTGATGCTGGTTGCCAAGCTCTCACGCTTAAAGGGTTTGGATGCCAGTATTGAGGCCGACATCGTAGCGGGGTTACAGGCATTACCGTCCCGGATCGAGCAAATGTTGAGTCAGGACAAACGCATTGAGGCACTGGCAGAAGATTTCAGTGACAAAGCTGCAGCGCTGTTCTTGGGCCGCGGGGATCAATACCCTATCGCACTCGAAGGCGCTCTGAAATTGAAGGAAATCTCTTACATCCATGCCGAGGCATACGCAGCAGGCGAGCTTAAGCATGGTCCATTGGCTTTAATTGACGCCGACATGCCGGTCATTGTAGTGGCACCTAACAATGAGTTACTGGAGAAGTTGAAATCCAATATTGAGGAAGTGCGTGCACGGGGGGGCCAGCTCTACGTATTTGCGGATCAGGATGCCGGGTTTGTGAGTTCCGACAACATGCATATCATCGAGATGCCACACGTGGAAGAAGTGATCGCACCGATTTTTTACACCGTCCCGCTGCAGTTATTAGCTTACCACGTAGCGTTGATTAAAGGGACCGATGTGGATCAGCCGCGGAATTTGGCAAAGTCAGTTACTGTCGAATGATGTAGGCTGGAGCTGCTTCGTACCGTT

Homology to glmS ORF in *E. coli*

kanamycin resistance gBlock

TGTAGGCTGGAGCTGCTTCGtaccgTTCGTATAGCATACATTATACGAAGTTATggaataggaacttcaagatcccctcacgctgccgcaagcactcagggcgcaagggctgctaaaggaagcggaacacgtagaaagccagtccgcagaaacggtgctgaccccggatgaatgtcagctactgggctatctggacaagggaaaacgcaagcgcaaagagaaagcaggtagcttgcagtgggcttacatggcgatagctagactgggcggttttatggacagcaagcgaaccggaattgccagctggggcgccctctggtaaggttgggaagccctgcaaagtaaactggatggctttcttgccgccaaggatctgatggcgcaggggatcaagatctgatcaagagacaggatgaggatcgtttcgcatgattgaacaagatggattgcacgcaggttctccggccgcttgggtggagaggctattcggctatgactgggcacaacagacaatcggctgctctgatgccgccgtgttccggctgtcagcgcaggggcgcccggttctttttgtcaagaccgacctgtccggtgccctgaatgaactgcaggacgaggcagcgcggctatcgtggctggccacgacgggcgttccttgcgcagctgtgctcgacgttgtcactgaagcgggaagggactggctgctattgggcgaagtgccggggcaggatctcctgtcatctcaccttgctcctgccgagaaagtatccatcatggctgatgcaatgcggcggctgcatacgcttgatccggctacctgcccattcgaccaccaagcgaaacatcgcatcgagcgagcacgtactcggatggaagccggtcttgtcgatcaggatgatctggacgaagagcatcaggggctcgcgccagccgaactgttcgccaggctcaaggcgcgcatgcccgacggcgaggatctcgtcgtgacccatggcgatgcctgcttgccgaatatcatggtggaaaatggccgcttttctggattcatcgactgtggccggctgggtgtggcggaccgctatcaggacatagcgttggctacccgtgatattgctgaagagcttggcggcgaatgggctgaccgcttcctcgtgctttacggtatcgccgctcccgattcgcagcgcatcgccttctatcgccttcttgacgagttcttctgagcgggactctggggttcgaaatgaccgaccaagcgacgcccaacctgccatcacgagatttcgattccaccgccgccttctatgaaaggttgggcttcggaatcgttttccgggacgccggctggatgatcctccagcgcggggatctcatgctggagttcttcgcccaccccagcttcaaaagcgctctATAACTTCGTATAGCATACATTATACGAAcggtaAACTGCAGGTCGACGGATCCCCGGAATgtttttaatcaaacatcctgccaactccatgtgacaaaccgtcatcttcggctactttttctctgtcacagaatgaaaatttttctgtcatctcttcgtt

LoxP LE

Kanamycin resistance gene

LoxP RE

Homology to sequence downstream of glmS ORF in *E. coli*

aceE gBlock sequence

CGACGCGGCTGAAGGTAAAAACATCGCGCACCAGGTTAAGAAAATGAACATGGACGGTGTGCGTCATATCCGCGACCGTTTCAATGTGCCGGTGTCTGATGCAGATATCGAAAAACTGCCGTACATCACCTTCCCGGAAGGTTCTGAAGAGCATACCTATCTGCTGGCGCAACGTCAAAAATTACACGGCTACCTGCCTTCTCGCCAACCAAACTTTACTGAGAAACTTGAGTTACCCAGCTTGCAGGATTTCGGTGCCTTGTTAGAGGAGCAATCAAAGGAAATTTCAACGACTATCGCTTTTGTGCGCGCTCTGAACGTCATGCTTAAAAACAAATCTATCAAGGATCGCTTGGTCCCCATCATCGCAGATGAGGCACGCACCTTCGGAATGGAAGGTCTTTTCCGCCAGATCGGTATCTACTCTCCTAACGGTCAACAATATACACCGCAGGACCGTGAGCAGGTCGCATACTATAAAGAAGATGAAAAAGGGCAGATCCTTCAGGAAGGGATCAACGAGCTGGGCGCGGGCTGTTCCTGGCTGGCCGCCGCAACTTCGTACTCCACGAATAACCTGCCGATGATTCCATTTTATATTTACTACTCCATGTTTGGTTTCCAGCGTATCGGTGACCTGTGCTGGGCTGCGGGAGACCAGCAAGCCCGTGGGTTTTTGATCGGGGGGACAAGTGGCCGTACTACGCTGAACGGGGAAGGCCTTCAACACGAGGACGGCCATTCTCACATTCAGTCTTTAACCATTCCGAACTGCATTAGTTACGACCCTGCATATGCCTACGAGGTGGCCGTCATTATGCACGATGGGTTGGAGCGCATGTATGGGGAAAAGCAAGAGAATGTCTATTATTACATTACTACTTTGAACGAGAACTATGCCATGCCAGCAATGCCTGAGGGTGCTGAGGAGGGAATTCGCAAGGGTATCTATAAGCTTGAGACAATTGAAGGCTCAAAAGGAAAGGTACAGTTATTAGGATCTGGGAGTATTCTTCGCCATGTTCGTGAGGCCGCCGAGATCCTTGCCAAGGACTACGGTGTCGGTTCGGATGTCTACAGCGTGACGTCCTTCACCGAATTGGCCCGTGACGGTCAAGATTGTGAACGCTGGAATATGTTGCACCCATTGGAAACGCCACGCGTACCGTACATTGCTCAAGTGATGAACGACGCGCCGGCGGTTGCCAGCACTGATTATATGAAATTATTTGCTGAACAAGTTCGTACTTACGTACCTGCAGATGACTATCGCGTTTTGGGAACCGATGGCTTTGGTCGTTCAGATAGCCGTGAAAATCTGCGCCGTTATTTCGAGGTGGACGCATCTTATGTTGTGGTCGCAGCGTTAGGCGAATTAGCTAAGCGCGGTGAAATTGATAAGAAAGTGGTTGCTGACGCGATTGCCAAATTTAATATTGATGCAGATAAAGTAAATCCGCGTTTAGCCTAAGAGCTGCTTCGtaccgTTCGTATAGCA

Figure S1 – gBlock sequences for construction of glmS and aceE targeting casettes.

Table S1 – Oligonucleotide sequences

| Primer | Sequence |
| --- | --- |
| glmS_seq_F | CGGGCATACAGGTTGACCGAC |
| glmS_seq_R | AGTCTTCAGTCTGATTTAAATAAGCG |
| glmS_6Ala_F | TGTTGATGCGGAAGGTCATATG |
| kanR_shortened_F | GAGCTGCTTCGtaccgTTCG |
| KanR_glmS_DS_R | aacgaagagatgacagaaaaattttc |
| AceE_F | CGACGCGGCTGAAGGTAAA |
| KanR_aceE_DS_R | AGCCCCGATGTCCGGTACTTTGATTTCGATAGCCATtattcttttacctc CCGTCGACCTGCAGTTtacc |
| AceE_seq_F | TGGTGCGTACGTTCGTGAACAC |
| AceE_seq_R | ATCTCGGTGATTTCAACTTCATC |


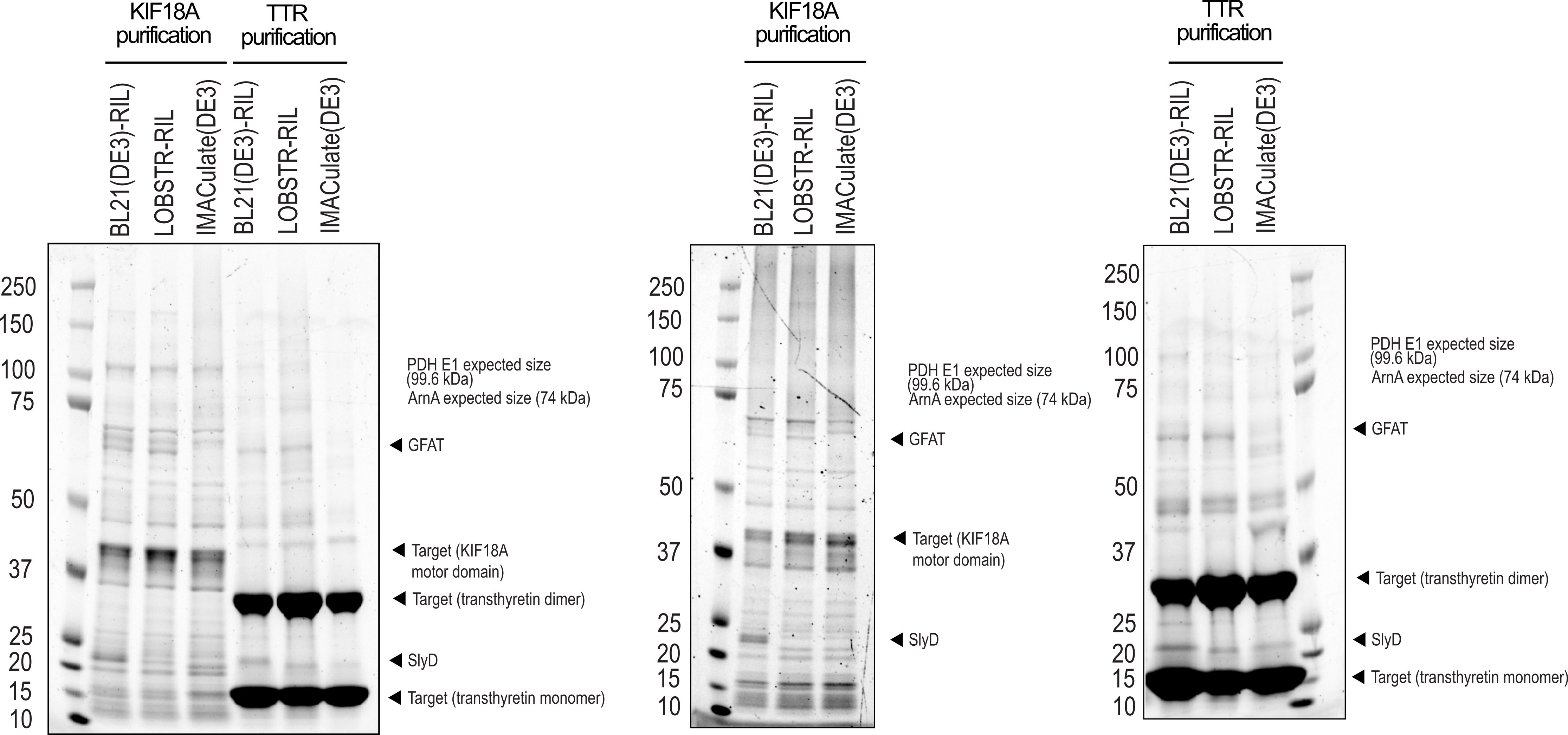


Figure S2. Biological repeat experiments of nickel IMAC purifications of KIF18A motor domain and transthyretin from IMACulate(DE3) show reduced GFAT impurity relative to BL21(DE3)-RIL and LOBSTR-RIL. Protein eluted from Ni-NTA resin was run on 4-12% BisTris SDS-PAGE gels (Thermo Fisher Scientific) using 1xMOPS buffer. Gels were stained for 15 min using InstantBlue Commassie dye (Abcam) and destained in deionised water overnight. Gels were imaged on a Chemidoc (BioRad) using Epi-Red setting for enhanced signal to noise. SlyD impurities was also observed to be reduced in KIF18A motor domain purification and TTR purification for IMACulate(DE3) and LOBSTR-RIL relative to BL21(DE3)-RIL.


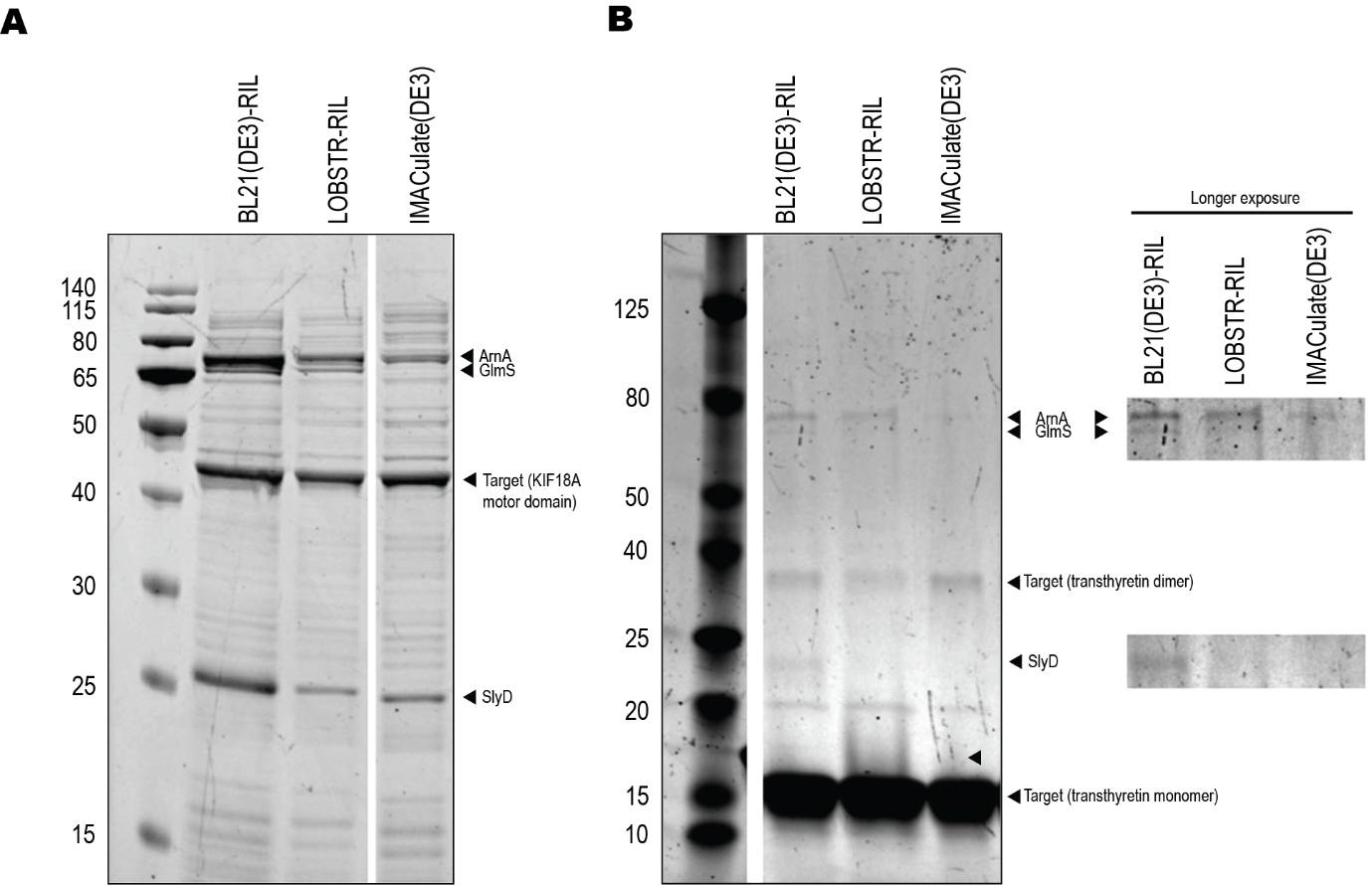


Figure S3. Nickel IMAC purifications of KIF18A motor domain (A) and transthyretin (B) from IMACulate(DE3) show reduced GFAT impurity relative to BL21(DE3)-RIL and LOBSTR-RIL. Protein eluted from Ni-NTA resin was run on 4-12% BisTris SDS-PAGE gels (Thermo Fisher Scientific) using 1xMOPS buffer. Gels were stained for 15 min using InstantBlue Commassie dye (Abcam) and destained in deionised water overnight. Gels were imaged on a Chemidoc (BioRad) using Epi-Red setting for enhanced signal to noise. SlyD and ArnA impurities was also observed to be reduced in KIF18A motor domain purification and TTR purification for IMACulate(DE3) and LOBSTR-RIL relative to BL21(DE3)-RIL.
